# Viable tendon neotissue from adult adipose-derived multipotent stromal cells

**DOI:** 10.3389/fbioe.2023.1290693

**Published:** 2024-01-08

**Authors:** Takashi Taguchi, Mandi Lopez, Catherine Takawira

**Affiliations:** Laboratory for Equine and Comparative Orthopedic Research, School of Veterinary Medicine, Veterinary Clinical Sciences Department, Louisiana State University, Baton Rouge, LA, United States

**Keywords:** bioengineering, ligament, stem cells, bioreactor, tissue regeneration, *de novo* tissue generation, equine

## Abstract

**Background:** Tendon healing is frequently prolonged, unpredictable, and results in poor tissue quality. Neotissue formed by adult multipotent stromal cells has the potential to guide healthy tendon tissue formation.

**Objectives:** The objective of this study was to characterize tendon neotissue generated by equine adult adipose-derived multipotent stromal cells (ASCs) on collagen type I (COLI) templates under 10% strain in a novel bioreactor. The tested hypothesis was that ASCs assume a tendon progenitor cell-like morphology, express tendon-related genes, and produce more organized extracellular matrix (ECM) in tenogenic versus stromal medium with perfusion and centrifugal fluid motion.

**Methods:** Equine ASCs on COLI sponge cylinders were cultured in stromal or tenogenic medium within bioreactors during combined perfusion and centrifugal fluid motion for 7, 14, or 21 days under 10% strain. Viable cell distribution and number, tendon-related gene expression, and micro- and ultra-structure were evaluated with calcein-AM/EthD-1 staining, resazurin reduction, RT-PCR, and light, transmission, and scanning electron microscopy. Fibromodulin was localized with immunohistochemistry. Cell number and gene expression were compared between culture media and among culture periods (*p* < 0.05).

**Results:** Viable cells were distributed throughout constructs for up to 21 days of culture, and cell numbers were higher in tenogenic medium. Individual cells had a round or rhomboid shape with scant ECM in stromal medium in contrast to clusters of parallel, elongated cells surrounded by highly organized ECM in tenogenic medium after 21 days of culture. Transcription factor, extracellular matrix, and mature tendon gene expression profiles confirmed ASC differentiation to a tendon progenitor-like cell in tenogenic medium. Construct micro- and ultra-structure were consistent with tendon neotissue and fibromodulin was present in the ECM after culture in tenogenic medium.

**Conclusion:** Long-term culture in custom bioreactors with combined perfusion and centrifugal tenogenic medium circulation supports differentiation of equine adult ASCs into tendon progenitor-like cells capable of neotissue formation.

## Introduction

Tendinopathy and desmitis comprise a large majority of musculoskeletal injuries that are responsible for up to 72% of lost training days and 14% of early retirements by equine athletes (Rossdale et al., 1985; Olivier et al., 1997; Lam et al., 2007). Superficial digital flexor tendinopathy and suspensory ligament desmitis are the most common, comprising 46% of all limb injuries (Williams et al., 2001; Bertuglia et al., 2014). The predominant type of tendon and ligament injury varies among disciplines, but all equine companions can be impacted. Strain induced injuries are common in the equine suspensory apparatus including the suspensory ligament, superficial digital flexor tendon, and deep digital flexor tendon (O’Sullivan, 2007). Many acute and chronic tendon and ligament lesions are thought to result from focal accumulation of microtrauma and poorly organized repair tissue that can coalesce into large lesions and predispose to spontaneous rupture in numerous species (Kannus and Jozsa, 1991).

Diagnosis is usually a combination of physical examination and ultrasound imaging (Dyson et al., 2018). Treatments vary widely and can range from rest with anti-inflammatory drugs, cold therapy, and pressure bandaging to intralesional therapies and extracorporeal shock wave treatment (Bostrom et al., 2022; Giunta et al., 2019; S; Witte et al., 2016). Intralesional regenerative treatments such as platelet rich plasma, stem cells, and genetic material have been applied with variable success (Aimaletdinov et al., 2020; Geburek et al., 2017; Kovac et al., 2018; Witte et al., 2011). Short-term outcomes of these treatments are favorable. However, poor, or abnormal tissue repair contributes to a reinjury rate in horses as high as 67% within 2 years (Dyson, 2004; Marr et al., 1993). To date, there is no single gold standard to promote healing of ligament and tendon lesions.

There are four recognized stages of tendon and ligament healing: an acute inflammatory phase, a subacute reparative phase, a collagen phase, and a chronic remodeling phase. Low cell numbers and metabolic activity, limited blood supply, and failure of endogenous tenocytes and ligamentocytes to migrate to the injury site affect all stages of healing and contribute to poor tissue healing capacity in adult animals (Longo et al., 2007; Sakabe and Sakai, 2011). Research confirms enhanced healing capacity of neonatal tendon over that of adults since early fibrous scar tissue is replaced with normal tendon by endogenous tenocytes recruited by TGF-β signaling (Howell et al., 2017; Kaji et al., 2020).

Autologous tenocyte implantation is one mechanism to deliver endogenous cells to the site of tendon or ligament injury in adult animals and humans (Chen et al., 2011; Wang et al., 2013; Wang et al., 2005); however, the therapy is limited by few harvest sites and harvest morbidity, and it is not practical in horses. Administration of exogenous adult multipotent stromal cells (MSCs) is reported to augment natural healing in naturally-occurring and experimentally-induced equine tendon and ligament injuries (Smith et al., 2013; Van Loon et al., 2014; Geburek et al., 2017; Romero et al., 2017; Carlier et al., 2023). Results are mixed, in part due to differences among cell isolates, lesions, individual healing capacity, and low engraftment of exogenous cells (<0.001%) (Reed and Leahy, 2013; Geburek et al., 2017). Further, there is evidence that an inflammatory environment may impede differentiation of MSCs, and the cells may assume an abnormal phenotype leading to unwanted side effects (Harris et al., 2004; Fahy et al., 2014).

The delivery of cells on scaffold matrix, often made of natural and/or synthetic polymers, is reported to improve cellular retention at the site of implantation (Pillai et al., 2017; Rashedi et al., 2017; Vadaye Kheiry et al., 2021; Zhao et al., 2010). Collagen is the most abundant natural polymer in the body and a common material for tissue engineering templates due to inherent biocompatibility (Xie et al., 2013). Collagen type I (COLI) comprises 60%–80% of tendon and ligament structure, and there are numerous commercially available, FDA-approved formulations (Cockerham and Hsu, 2009; Longo et al., 2010; Snedeker and Foolen, 2017; Meyer, 2019). Scaffolds composed of COLI are routinely used for delivery and retention of stem cells in tendon and ligament tissue (Ma et al., 2019). Published information confirms that COLI matrix supports differentiation of equine MSCs into diverse tissue lineages (Xie et al., 2013; Muller et al., 2016; Duan et al., 2017). Additionally, evidence suggests differentiation of MSCs into lineages native to the site of injection prior to implantation is essential (Shojaee, Parham, 2019). Pre-implantation differentiation of cells into tenocytes is reported to minimize the risk of ectopic bone and cartilage formation as well as tumor formation at the site of injection (Lui et al., 2011). Recently, injection of tenogenically differentiated allogeneic equine MSCs into naturally occurring tendon lesions resulted in a lower reinjury rate (18%) than conventional treatments (44%) 24 months post-treatment (Beerts et al., 2017). Regardless of the specific target tissue, current knowledge supports that MSCs that are induced to assume characteristics of native tissue lineage and embedded in scaffold matrix prior to implantation have better engraftment and promote more robust tissue healing than undifferentiated primary cell isolates (Aurich et al., 2009).

In the study reported here, tendon neotissue was created by culturing equine adult adipose-derived MSCs (ASCs) on COLI templates. The templates were maintained under 10% strain with combined perfusion and centrifugal culture medium motion within custom perfusion bioreactors. The hypothesis was that ASCs assume a tendon progenitor cell-like morphology, express tendon-related genes, and produce more organized extracellular matrix in tenogenic versus stromal medium with perfusion and centrifugal fluid motion for 7, 14, or 21 days. Generation of viable tendon neotissue implants has the potential to augment contemporary therapies for equine tendinopathy. Longitudinal evaluation of constructs from shortly after fabrication to up to 21 days of culture is vital to establishing the process and time course of *de novo* equine tendon tissue generation for preclinical testing and clinical translation.

## Materials and methods

### Study design

**FIGURE 1:**
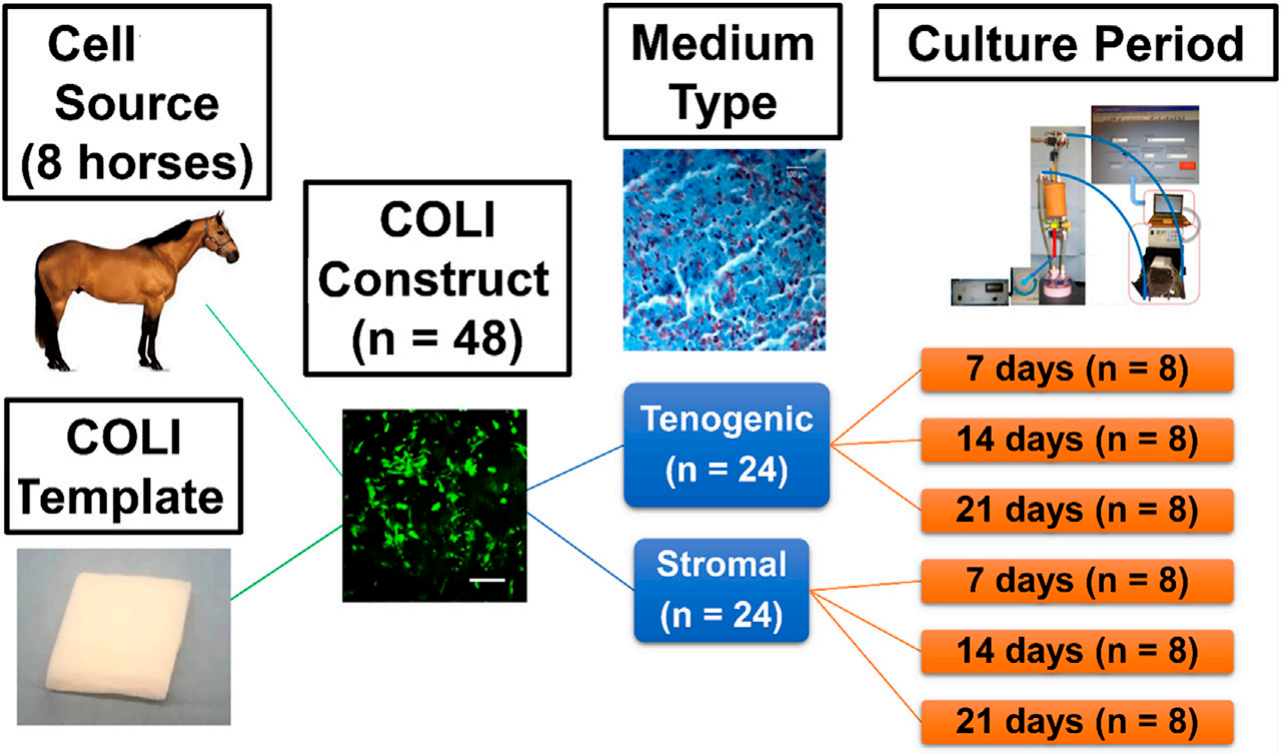
Study design schematic.

Equine ASC-COLI constructs were created by addition of 1.0 × 10^6^ cells/cm^3^ (P2) from individual donors (4 geldings, 4 mares, body condition score 4–7, 5–21 years, 425–500 kg) to culture medium in separate, custom-designed perfusion bioreactor chambers that each contained a cylinder of commercially available bovine COLI (Avitene™ Ultrafoam™ Collagen Sponge, Davol Inc., Warwick, RI) (*n* = 48 constructs, 3 construct pairs/donor). One-half of the constructs from each donor were cultured in stromal or tenogenic medium (*n* = 3 medium/donor) within individual perfusion bioreactors for 7, 14, or 21 days (*n* = 1 donor/medium/time point) under 10% static strain (Figure 1). Construct gross appearance was documented with digital imaging prior to sample collection from the upper, middle, and lower regions of the construct relative to bioreactor orientation for outcome measures. Viable cell distribution and number, tendon-related gene expression, and micro- and ultra-structure were evaluated with calcein-AM/EthD-1 staining, resazurin reduction, RT-PCR, and light-as well as transmission and scanning electron microscopy. The deposition of fibromodulin within constructs was localized with immunohistochemistry. Fibromodulin is a key regulator of tendon fibril maturation and it is most prevalent in the later stages of embryonic fibrillogenesis (Banos et al., 2008; Zhao et al., 2023).

### Primary cell isolates

No horses were euthanized for this study. All tissues for primary cell isolation were collected immediately post-mortem from horses euthanized as part of an approved study protocol (IACUCAM- 21–141) that was unrelated to this study. About 45 mL of subcutaneous adipose tissue was aseptically harvested via sharp dissection from the supragluteal region of 4 adult geldings and 4 mares (Vidal et al., 2007). Harvested tissues were placed in sterile 50 mL conical tubes (Nunc™, Thermo Fisher Scientific, Waltham, MA) for transport. Donor inclusion criteria were: 1) 425–500 kg; 2) 5–21 years; 3) no acute or chronic systemic illness; 4) mare or gelding; 5) body condition score 4–7. The stromal vascular fraction was isolated as previously described within 2 h of harvest (Vidal et al., 2007). Briefly, adipose tissue was minced and mixed with an equal volume of phosphate buffered saline (PBS, PBS 1X, Thermo Fisher Scientific). The mixture was allowed to separate into two phases, and the infranatant was digested for 2 h at 37°C in an equal volume of PBS with 1% bovine serum albumin (BSA, Sigma Aldrich, Co, Saint Louis, MO) and 0.1% type I collagenase (Worthington Biochemical, Lakewood, NJ). After addition of 1% BSA, the mixture was centrifuged (2.6 × 10^2^ g, 5 min, 4°C). The resulting stromal vascular fraction pellet was resuspended in PBS and centrifuged (2.6 × 10^2^ g, 5 min). The pellet was resuspended in stromal medium (Dulbecco’s modified eagle medium (DMEM)-Ham F12 (HyClone Laboratories, LLC, Logan, UT), 10% fetal bovine serum (FBS, HyClone Laboratories), 1% antibiotic/antimycotic solution (HyClone Laboratories). Viable cell numbers were quantified with methylene blue (methylene blue hydrate, Sigma Aldrich) staining and a hemocytometer (Hausser Scientific™ Bright-Line™ Counting Chamber, Fisher Scientific).

Isolated cells were cultured in 10 cm culture dishes (CellStar^®^, VWR, Radnor, PA) at 5 × 10^3^ cells/cm^2^ with stromal medium that was refreshed after 24 h and then every 2-3 days (5% CO_2_, 37°C, 90% humidity). Cells were detached [25% trypsin (HyClone Laboratories)] and passaged at 80% confluence. Subsequently, P0 cells (10^6^ cells/mL) in cryopreservation medium [80% FBS, 10% DMEM-Ham F12, 10% DMSO (Thermo Fisher Scientific)] within cryopreservation vials (Fisherbrand™ Externally and Internally Threaded Cryogenic Storage Vials, Thermo Fisher Scientific) were cooled to −80°C in a freezing container (Corning^®^ CoolCell™ Freezer Container, Sigma Aldrich) and then transferred to liquid nitrogen (−150°C). Samples in cryopreservation vials were thawed in a water bath (37°C), and cryopreservation medium was removed by resuspension of cell pellets in PBS after centrifugation (2.6 × 10^2^ g, 5 min) 3-4 times. Cells were subcultured to P1 in stromal medium as described above. Passage 2 cells on COLI templates were cultured in tenogenic (DMEM-high glucose (HyClone Laboratories), 1% FBS, 10 ng/mL transforming growth factor (TGF)-β1 (Shenandoah Biotechnology, Inc., Warminster, PA), 50 μM L-ascorbic acid 2-phosphate sesquimagnesium salt hydrate (Sigma Aldrich), 0.5 μg/mL insulin (Sigma-Adrich), 1% antibiotic/antimycotic) (Theiss et al., 2015) or stromal medium [(Dulbecco’s modified Eagle medium (DMEM)-Ham F12 (Hyclone Laboratories), 10% fetal bovine serum (FBS, HyClone Laboratories), 1% antibiotic/antimycotic solution (HyClone Laboratories)].

### Perfusion bioreactor system

Templates were composed of a commercially available COLI sponge consisting of a partial hydrochloric acid salt of purified bovine corium (Avitene™ Ultrafoam™ Collagen Sponge, Davol Inc.) that is approved for use as microfibrillar collagen hemostat by the US Food and Drug Administration (Zhou et al., 2016; Cziperle, 2021). The sponge is porous, pliable, water insoluble and bioabsorbable, and it is produced by lyophilization of a slurry of water and purified collagen. The manufacturing process permits noncovalent attachment of hydrochloric acid to amine groups on the collagen molecules and preserves their native morphology.

**FIGURE 2:**
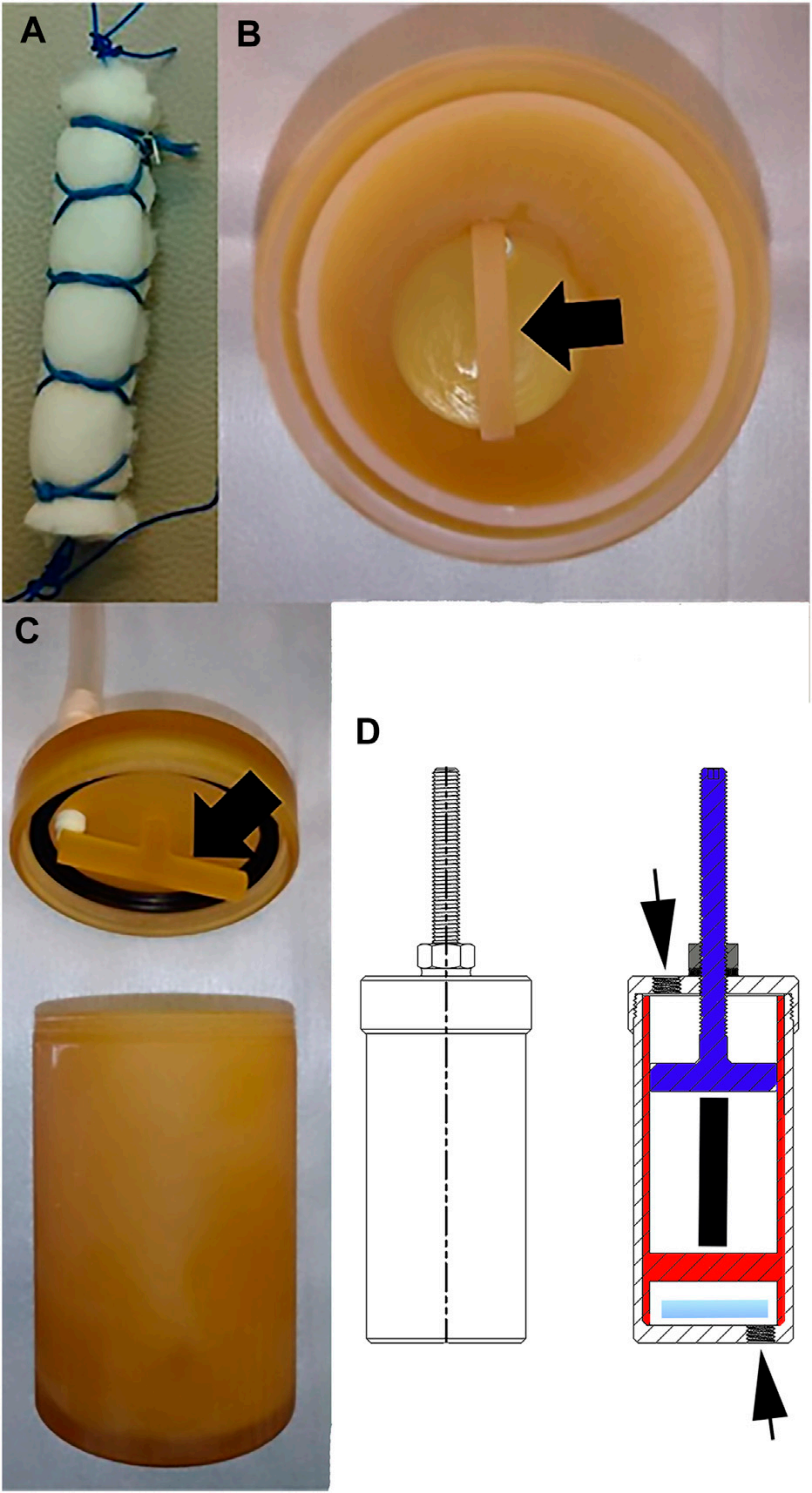
Bioreactor. Photographs of a COLI template **(A)**, the immobile horizontal bar (black arrow) at the lowest end of the frame insert within a bioreactor chamber **(B)**, the adjustable horizontal bar (black arrow) on the lid of the bioreactor chamber **(C)**, and drawings of the outside (left) and inside (right) of the bioreactor **(D)**. The adjustable top bar attached to the bioreactor lid (blue) and secured with a nut (gray) on a rubber washer (horizontal black line beneath the washer), the insert (red) with the lower bar, the stir bar (light blue), the position of the construct within the bioreactor (black vertical line) and the ports on the cap and the bottom of the bioreactor (black arrows) are illustrated.

For each template, a COLI sponge section, 6.0 × 4.0 × 1.0 cm (length × width × height), was rolled into a column with a diameter of 1.0 cm and height of 6.0 cm that was surrounded by a finger trap composed of #0 polydioxanone suture (PDS^®^ II, Ethicon, Inc., Somerville, NJ) (Figure 2A). There were 1 cm long loops on each end. The bioreactor was a custom design by the authors that was produced by stereolithography out of liquid resin (SOMOS^®^ WaterShed^®^ XC 11122, Stratasys, Waltham, MA) by a commercial printing company (Proto Labs, Inc., Maple Plain, MN). A frame with an immobile cross beam on the lowest end fit within a single bioreactor chamber measuring 80 × 45 mm (height × diameter) (Figures 2B–D). The bioreactor lid was secured to the top of the chamber with matching threads. A threaded, vertically adjustable cross beam attached to the upper lid with matching threads extended downward into the bioreactor chamber within the frame insert. The distance of the bar from the lid (and the lower beam on the insert) was adjusted by turning the beam to advance toward or away from the lid, and the position was fixed with a nut on the top of the lid. The space between the nut and the lid was sealed with sterile rubber gasket material. Constructs were secured to the upper beam on the lid and to the lower beam on the frame with the suture loops on each end. The distance between the beams was initially set at the correct distance for a 10% construct strain based on the length of the construct with no tension applied as determined with an electronic caliper (Mitutoyo #500–196, Mitutoyo Corp., Japan). When medium was exchanged every 7 days, the height of the upper beam was adjusted if necessary to maintain 10% strain based on the construct length determined at that time. A magnetic stir bar (2.5 × 0.7 cm) was placed in the chamber beneath the lowest bar of the frame insert.

**FIGURE 3:**
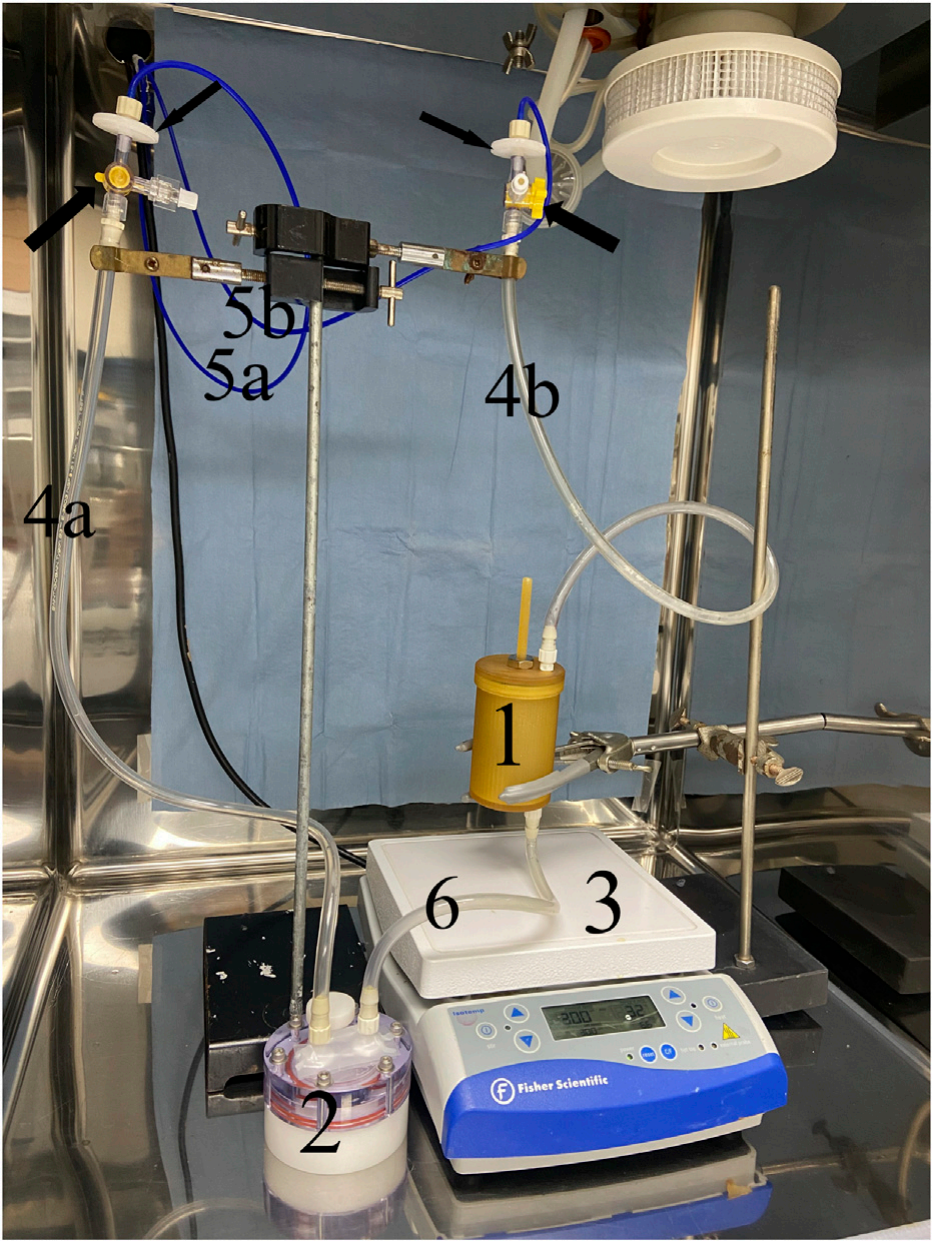
Perfusion bioreactor system. Medium perfusion was driven by a computer-controlled peristaltic pump connected to the bioreactor chamber (1) and a medium reservoir (2). The pump was attached with 1.0 mm tubing (5a) to a 0.22 µm syringe filter which was connected to a segment of 4.8 mm tubing (4a) attached to one port on the upper surface of a 10 mL medium reservoir (2). Another port on the upper surface of the medium reservoir was attached to the lower port of the bioreactor chamber with 4.8 mm tubing (6). The pump was also connected with 1.0 mm tubing (5b) to another 0.22 µm syringe filter (small black arrow) which was connected with a 3-way stopcock (large black arrow) 4.8 mm tubing (4b) attached to the upper port on the bioreactor chamber (1). A magnetic stir bar at the lowest end of the bioreactor chamber driven by a stir plate (3) provided centrifugal fluid motion.

Medium perfusion was driven by a computer-controlled peristaltic pump (Ismatec model ISM404b, Huiyu Weiye (Beijing) Fluid Equipment Co., Ltd., Beijing, China) connected to the bioreactor chamber and a medium reservoir (Figure 3). Specifically, tubing (1.0 mm inner diameter; Tygon^®^; Compagnie de Saint-Gobain, Courbevoie, Centre, France) extended from the pump to a 0.22 µm sterile syringe filter (MilliporeSigma™, Thermo Fisher Scientific) which was connected with a 3-way stopcock to 55 cm of additional tubing (4.8 mm inner diameter; Tygon^®^, Compagnie de Saint-Gobain) connected to a port on the bioreactor. The pump was separately attached with tubing (1.0 mm inner diameter; Tygon^®^, Compagnie de Saint-Gobain) to a 0.22 µm sterile syringe filter (MilliporeSigma™, Thermo Fisher Scientific) which was connected to another 55 cm segment of tubing (4.8 mm inner diameter; Tygon^®^, Compagnie de Saint-Gobain) attached to one port on the upper surface of a 10 mL medium reservoir (High Aspect Ratio Vessel, Synthecon, Inc., Houston, TX). Another port on the upper surface of the medium reservoir was attached to the lower port of the bioreactor chamber with 10 cm of tubing (4.8 mm inner diameter; Tygon^®^, Compagnie de Saint-Gobain). The reservoir provided medium oxygenation via a flat, silicone rubber gas transfer membrane.

A fluid flow rate of 10 mL/min was maintained by a computer program (LabView^™^, National Instruments, Austin, TX), and the direction of fluid flow was reversed when it reached the upper and lower ends of the tubing between the bioreactor lid and the syringe filter. Centrifugal medium motion within the chamber was generated with the magnetic stir bar beneath the frame in the chamber (300 rpm) that was driven by a stir plate (Isotemp™, Thermo Fisher Scientific) positioned beneath the chamber system. The fluid flow was both ingress-egress and centrifugal. Fluid flow rate and stir speed were based on previous work with custom perfusion and spinner flask bioreactors as well as progressive iterations of the current system to optimize viable cell distribution in the scaffold (Xie et al., 2013; Duan et al., 2017; Taguchi et al., 2021). All bioreactor system parts were sterilized with ethylene oxide prior to assembly and use. The perfusion system was maintained in a CO_2_ incubator (5% CO_2_, 37°C) for the duration of the culture period.

A previously reported method was used to seed P2 ASCs on the COLI template (Taguchi et al., 2021). Specifically, a template was premoistened and added to the bioreactor after the system was filled with stromal or tenogenic culture medium. It was maintained in the incubator for 1 h with fluid motion. Subsequently, the fluid motion was paused. Cells (1 × 10^6^ cells/cm^3^ template) were added to the medium of the bioreactor chamber through the 3-way port on the tubing attached to the bioreactor lid, and fluid motion was restarted. Medium was exchanged every 7 days through the reservoir (Taguchi et al., 2021).

### Construct sample collection

Constructs were harvested after 7, 14, or 21 days of culture. To ensure collection of representative samples from the entire construct length and width, constructs were gently unrolled following suture removal. The long axis of each construct rectangle was divided into three equal regions (approximately 2 cm each) that corresponded to the highest, middle, and lowest point of the bioreactor. A 4.0 mm diameter biopsy punch (Integra™ Miltex™ Standard Biopsy Punches, Thermo Fisher Scientific) was used to collect full thickness samples from each region. Differences in cell viability staining among harvest regions were subjectively assessed. Samples from each region were combined for all other outcome measures.

### Viable cell distribution and relative cell number

To evaluate viable cell distribution, specimens were incubated in darkness with 4.0 μM calcein acetoxymethyl ester (Invitrogen™, calcein-AM, Thermo Fisher Scientific) and ethidium homodimer-1 (Invitrogen™ EthD-1, Thermo Fisher Scientific) in PBS at 20°C for 30 min to stain viable and nonviable cells, respectively. After incubation, specimens were washed with PBS, compressed between a glass slide and cover glass, and evaluated with a confocal laser microscope (TCS SP8, Leica, Wetzlar, Germany). Cell morphology and viability were assessed in multiple z planes to examine the full thickness of each specimen. Digital images were generated with a camera integrated into the microscope.

The number of viable cells in each specimen was indirectly quantified based on cell metabolic activity measured by resazurin reduction (alamarBlue™, Thermo Fisher Scientific). Individual biopsy samples were incubated in 100 μL of 50 μM resazurin at 37°C for 3 h. After incubation, 50 μL was mixed with 50 μL of PBS, and resorufin fluorescence was measured at an excitation wavelength of 540 nm and an emission wavelength of 590 nm using a microplate reader (SPARK^®^ Multimode Microplate Reader, Tecan, Männdorf, Switzerland). Fluorescence values were normalized to background fluorescence measured from resazurin solution incubated with no specimen.

### Gene expression

Specimens were digested at 37°C in 0.1% type I collagenase in PBS for 1 h, centrifuged (3 × 10^2^ g, 10 min, 4°C), and the supernatant discarded. One milliliter of TRI reagent^®^ (Sigma Aldrich) was immediately added to the precipitate, the solution homogenized by aspirating through an 18-gauge needle 30 times, and the homogenate centrifuged (2.1 × 10^4^ g, 15 min, 4°C). Total RNA was initially extracted with phenol-chloroform (Sigma Aldrich) and then with a commercially available kit (RNeasy^®^ Mini Kit, QIAGEN Sciences, Germantown, MD). One microgram of total RNA was used for cDNA synthesis (QuantiTect^®^ Reverse Transcription Kit, QIAGEN Sciences).

Equine-specific primers for tendon-related genes, *scleraxis* (*Scx*), *mohawk* (*Mkx*), *early growth response 1* (*Egr1*), *connective tissue growth factor* (*CTGF*), *lysyl oxidase* (*LOX*), *collagen 1a1* (*Col1a1*), *collagen 3a1* (*Col3a1*), *decorin* (*Dcn*), *elastin* (*Eln*), *tenascin-c* (*Tnc*), *biglycan* (*Bgn)*, *fibromodulin* (*Fbmd)*, *collagen 14a1* (*Col14a1*), and *truncated hemoglobin 4* (*THBS4),* were prepared using previously published sequences or designed with Primer-BLAST (Ye et al., 2012; Miyabara et al., 2014; Mohanty et al., 2014; Jacobson et al., 2015; Yang et al., 2019) (Table 1). The PCR cycles included an initial denaturation step (95°C, 15 min) followed by 40 denaturation cycles (94°C, 15 s), annealing (52°C, 30 s), and elongation (72°C, 30 s) using SYBR Green (QuantiTect^®^ SYBR^®^ Green PCR Kits, QIAGEN Sciences). Target gene expression in constructs cultured in tenogenic and stromal medium was normalized to the reference gene g*lyceraldehyde 3-phosphate dehydrogenase* (*GAPDH*). Fold change between constructs cultured in tenogenic versus stromal medium was calculated as 2^−ΔΔCT^. The relative gene expression for constructs cultured in stromal medium (reference) was set to 1 since when ΔΔCT is equal to 0 (no change), 2^0^ is equal to 1 (Livak and Schmittgen, 2001; Abate et al., 2009).

**TABLE 1:** Equine-specific primer sequences.

Gene		Sequence (5'->3')	Amplicon Length	Accession Number
*Scx* ^c^	Forward	TCT​GCC​TCA​GCA​ACC​AGA​GA	246	NM_001105150.1
	Reverse	AAA​GTT​CCA​GTG​GGT​CTG​GG		
*Mkx* ^c^	Forward	AGT​GGC​TTT​ACA​AGC​ACC​GT	217	XM_023632371.1
	Reverse	ACA​CTA​AGC​CGC​TCA​GCA​TT		
*Egr1* ^d^	Forward	CCT​ACG​AGC​ACC​TGA​CCT​CAG	241	XM_001502553.5
	Reverse	GAT​GGT​GCT​GAA​GAT​GAA​GTG​G		
*CTGF*	Forward	ACC​CGC​GTT​ACC​AAT​GAC​AA	140	XM_023651101.1
	Reverse	GGC​TTG​GAG​ATT​TTG​GGG​GT		
*LOX*	Forward	CAG​GCG​ATT​TGC​GTG​TAC​TG	301	XM_023617821.1
	Reverse	ACT​TCA​GAA​CAC​CAG​GCA​CT		
*Col1a1* ^c^	Forward	CAA​GAG​GAG​GGC​CAA​GAA​GA	261	XM_023652710.1
	Reverse	TCC​TGT​GGT​TTG​GTC​GTC​TG		
*Col3a1*	Forward	TCC​TGG​GGC​TAG​TGG​TAG​TC	255	XM_008508902.1
	Reverse	GGC​GAA​CCA​TCT​TTG​CCA​TC		
*Dcn* ^b^	Forward	TTATCAAAGTGCCTGGTG	204	XM_005606467.3
	Reverse	CATAGACACATCGGAAGG		
*Eln* ^d^	Forward	CTA​TGG​TGT​CGG​TGT​CGG​AG	247	XM_023655466.1
	Reverse	GGG​GGC​TAA​CCC​AAA​CTG​AG		
*Tnc*	Forward	TAC​TGA​TGG​GGC​CTT​CGA​GA	330	XM_023628745.1
	Reverse	AGC​AGC​TTC​CCA​GAA​TCC​AC		
*Bgn* ^a^	Forward	TGA​TTG​AGA​ACG​GGA​GCC​TGA​G	143	XM_023633175.1
	Reverse	TTT​GGT​GAT​GTT​GTT​GGT​GTG​C		
*Fbmd* ^b^	Forward	GCTTCTGCTGAGGGACAC	91	NM_001081777.1
	Reverse	GATTTCTGGGGTTGGGAC		
*Col14a1* ^b^	Forward	CTGGACGATGGAAGTGAG	215	XM_005613197.3
	Reverse	GTGACCCTGAACTGCTGC		
*THBS4*	Forward	ACG​TAA​ACA​CCC​AGA​CGG​AC	359	XM_023618094.1
	Reverse	CAC​CAA​CTC​GGA​GCC​TTC​AT		
*GAPDH* ^b^	Forward	GTGTCCCCACCCCTAACG	131	NM_001163856.1
	Reverse	AGTGTAGCCCAGGATGCC		

a (Jacobson et al., 2015).

b (Miyabara et al., 2014).

c (Mohanty et al., 2014).

d (Yang et al., 2019).

### Histological analysis

Specimens were fixed in 4% paraformaldehyde, serially dehydrated in increasing concentrations of ethanol and xylene, paraffin embedded, sectioned (5 μm) and stained with hematoxylin and eosin (H&E). Cell morphology, distribution, and extra cellular matrix were evaluated on digital images generated with a slide scanner (NanoZoomer S20, Hamamatsu Photonics, Shimokanzo, Iwata City, Shizuoka Pref., 438-0193, Japan) or with a light microscope (DM4500B, Leica) fitted with a camera (DFC480, Leica).

### Immunohistochemical fibromodulin staining

Paraffin sections on glass slides were incubated in PBST (0.1% Triton X-100 (Sigma Aldrich) in PBS) at 20°C for 10 min, in antigen retrieval buffer [100 mM Tris (Sigma Aldrich), 5% Urea (Sigma Aldrich) pH 9.5] at 121°C for 30 min, and then in blocking buffer (1% BSA and 22.5 mg/mL glycine (Sigma Aldrich) in PBST) at 20°C for 30 min. Subsequently, slides were incubated with rabbit anti-human fibromodulin (PA5-26250, Thermo Fisher Scientific) polyclonal antibody at concentration of 1:100 in incubation buffer (1% BSA in PBST) overnight at 4°C. Sections were washed with PBS at 20°C 3 times for 15 min each, then stained with goat anti-rabbit IgG conjugated with Alexa Fluor™ 488 (A11070, Thermo Fisher Scientific) at a concentration of 1:200 in incubation buffer for 1 h at 20°C. Following washing with PBS 3 times, nuclei were counter-stained with 4’,6-diamidino-2-phenylindole (DAPI, Thermo Fisher Scientific) at a concentration of 10 μM in PBS at 20°C for 10 min. Sections were washed with PBS once and mounted with mounting medium (Vectashield^®^ Antifade Mounting Medium, Vector Laboratories, Newark, CA) beneath a cover glass. Digital images were obtained at an excitation wavelength of 490 nm and an emission wavelength of 525 nm using a camera integrated into a confocal microscope (TCS SP8, Leica). Equine deep digital flexor tendon sections were stained as positive controls and construct sections were stained with secondary antibody alone as negative controls. Staining was assessed subjectively.

### Scanning electron microscopy (SEM)

Specimens were fixed in 2% paraformaldehyde and 1.25% glutaraldehyde in 0.1 M sodium cacodylate (CAC) buffer (pH 7.4) for 1 h at 25°C and transferred to buffer (3% glutaraldehyde in 0.1 M CAC buffer, pH 7.4) for 30 min. They were rinsed with washing buffer (5% sucrose in 0.1 M CAC buffer, pH 7.4), post-fixative buffer (1% osmium tetroxide in 0.1 M CAC buffer, pH 7.4), and water. Specimens were serially dehydrated, critical point dried, and sputter coated with gold. Digital images were created with a scanning electron microscope and camera at 15 kVp (Quanta 200, FEI Company, Hillsboro, OR).

### Transmission electron microscopy (TEM)

Specimens collected from constructs cultured in stromal or tenogenic medium for 21 days were fixed in 2% paraformaldehyde and 2% glutaraldehyde in 0.1 M PBS (pH 7.4) at 4°C overnight. They were washed 3 times in 0.1 M PBS for 30 min each, and post-fixed in 2% osmium tetroxide (OsO_4_) in 0.1 M PBS at 4°C for 3 h. They were dehydrated in graded ethanol, infiltrated with propylene oxide twice for 30 min each, and placed in a 70:30 mixture of propylene oxide and resin for 1 h followed by polymerization in 100% resin at 60°C for 48 h. Polymerized resins were sectioned (70 nm) with a diamond knife using an ultramicrotome (Ultratome Leica EM UC7, Leica), mounted on copper grids, and stained with 2% uranyl acetate at 20°C for 15 min. They were stained with lead stain solution at 20°C for 3 min. Images were obtained with a transmission electron microscope [JEM-1011, JEOL Ltd., Zhubei City, Hsinchu County 302,004, Taiwan (R.O.C.)] at an acceleration voltage of 80 kV.

### Statistical analysis

Individual data points are shown on scatter plots with mean ± standard error of the mean (SEM) indicated. Differences in RFU were evaluated with two-way ANOVA and post-hoc pairwise Tukey’s tests with treatment, culture period and their interaction as the fixed effects (JMP Pro 17.0.0, JMP Statistical Discovery LLC, Cary, NC). Differences in gene expression fold change from baseline (1) in constructs cultured in tenogenic medium for each culture period were determined with one sample t-tests for normally distributed results and Wilcoxon signed rank tests for non-normally distributed results (Prism, v7, GraphPad Software Inc., La Jolla, CA). Significance was set at *p* ≤ 0.05.

## Results

### Construct gross appearance

**FIGURE 4:**
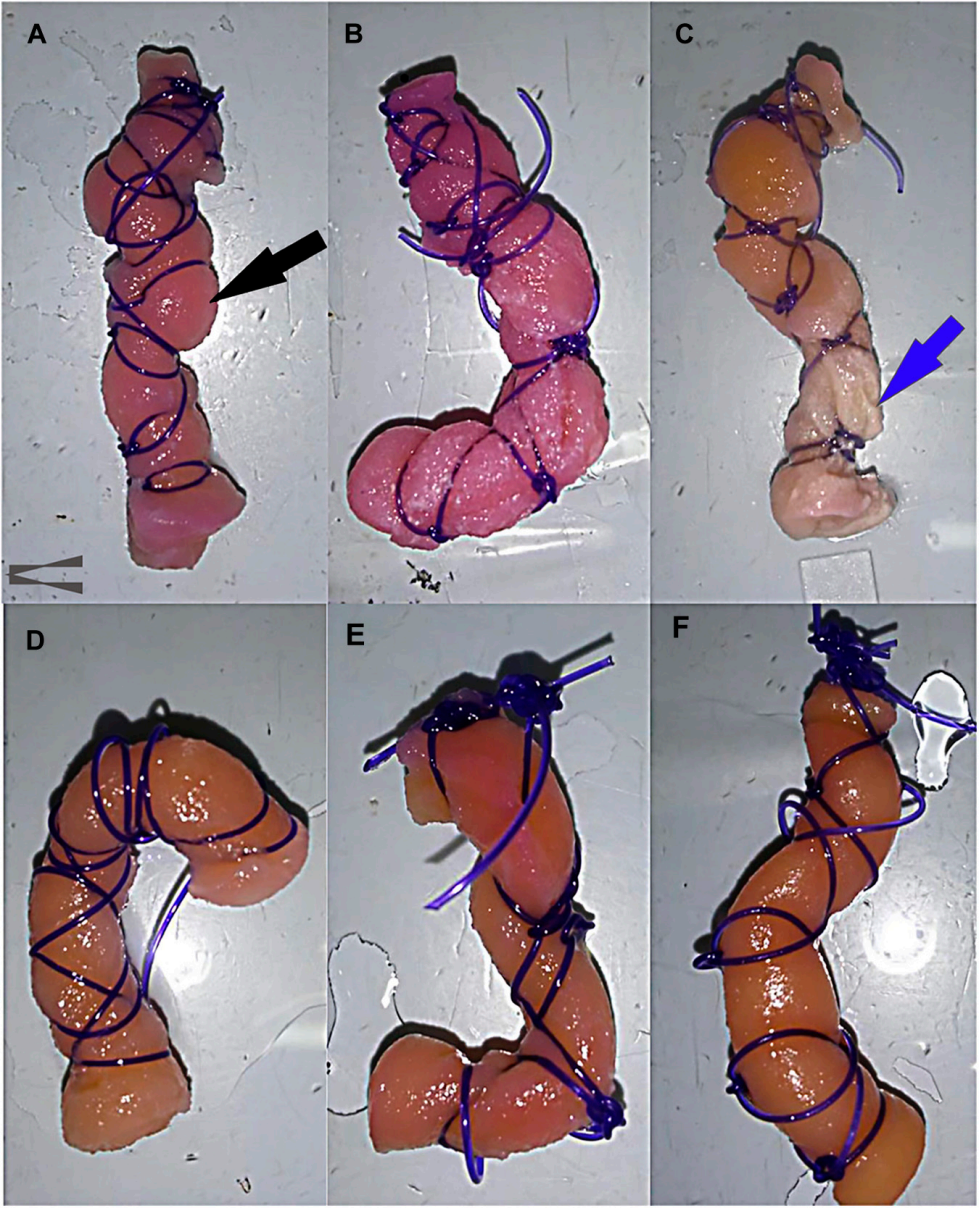
Gross appearance of COLI-ASC constructs. Photographs of untensioned constructs cultured in stromal **(A–C)** or tenogenic **(D–F)** medium for 7 **(A,D)**, 14 **(B,E)**, or 21 **(C,F)** days with construct bulging through the finger trap suture (black arrow) and construct erosion (blue arrow) indicated. The upper portion of each image corresponds to the upper end of the bioreactor.

After removal from the bioreactor and a thorough PBS rinse, the gross appearance of constructs cultured in stromal medium (Figures 4A–C) was different from those cultured in tenogenic medium (Figures 4D–F) beginning after 7 days of culture. The size of constructs cultured in stromal medium did not change much after 7 days of culture evidenced by construct bulging between finger trap suture loops (Figure 4A), like dry templates (Figure 2A). Notably, the template material alone does not expand when moistened (Zhou et al., 2016). After 14 days, there was no appreciable change in size, but the surface of the lower half of constructs cultured in stromal medium was lighter in color and roughened compared to the upper half (Figure 4B). After 21 days of culture, the upper half of the constructs were a tan color, the lower half an off-white color, and construct material was frequently missing at the lowest end (Figure 4C). Constructs cultured in tenogenic medium contracted after 7 days of culture based on loose finger trap suture loops. The constructs were a tan color with occasional red patches and surfaces were roughened (Figure 4D). After 14 days of culture in tenogenic medium, the constructs were a similar color to after 7 days, but had a smooth surface and had contracted more (Figure 4E). The constructs contracted such that the suture loops rarely contacted the template, more so in the upper half, were a solid tan color, and had an even smoother surface after 21 days of culture (Figure 4F). Additionally, the constructs were less compressible and the template layers more tightly adhered than those cultured in stromal medium for 21 days or uncultured template material.

### Viable cell distribution and relative cell number

**FIGURE 5:**
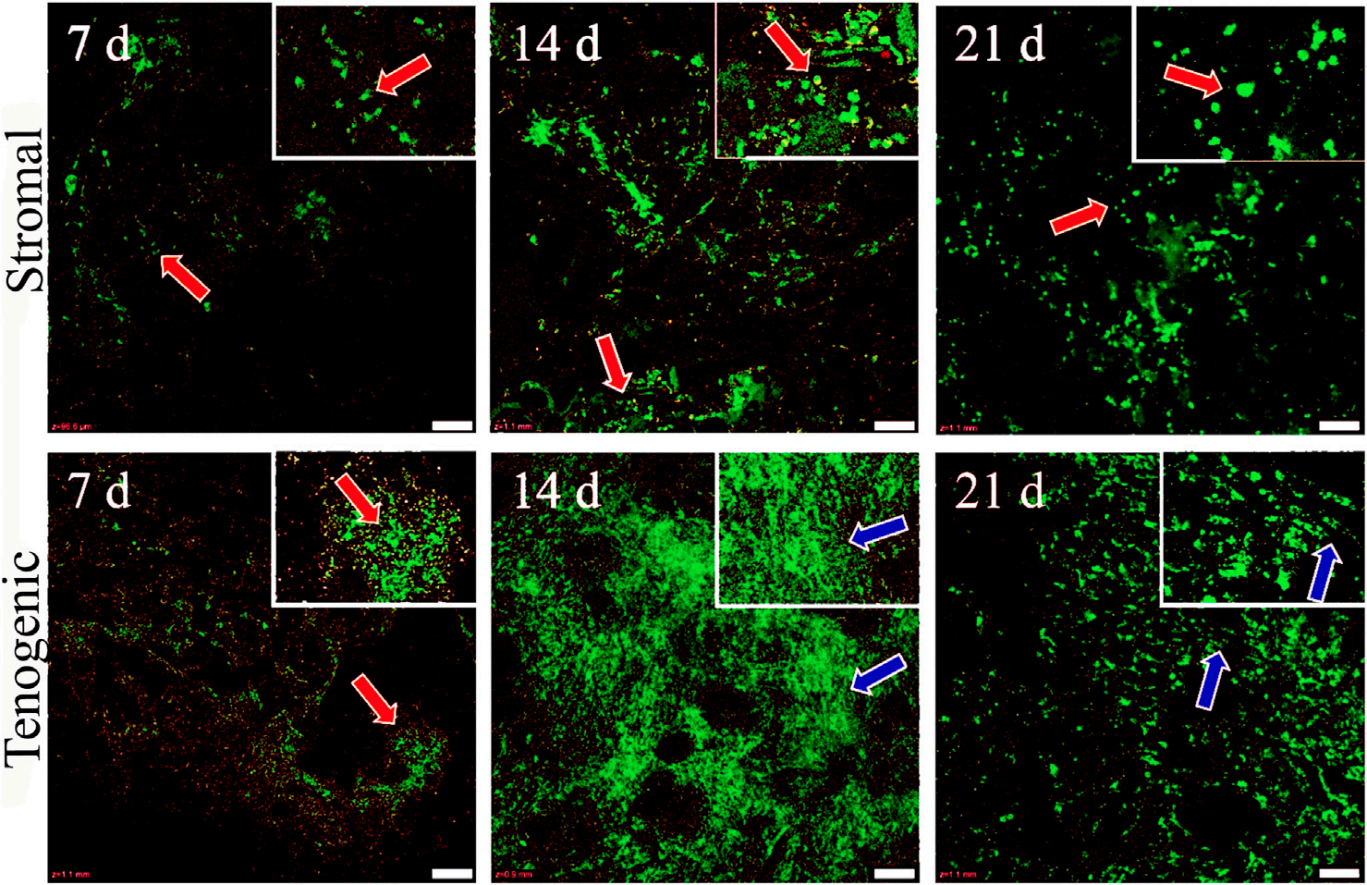
Viable cell distribution in COLI-ASC constructs. Photomicrographs of constructs cultured in stromal (upper) or tenogenic (lower) medium for 7, 14, or 21 days with green viable and red nonviable cells shown. Cells with spherical (orange arrows) and elongated (blue arrows) morphology are indicated. Insets at the top right corners are enlargements of areas around the arrows in the larger images. Scale bars = 100 μm.

**FIGURE 6:**
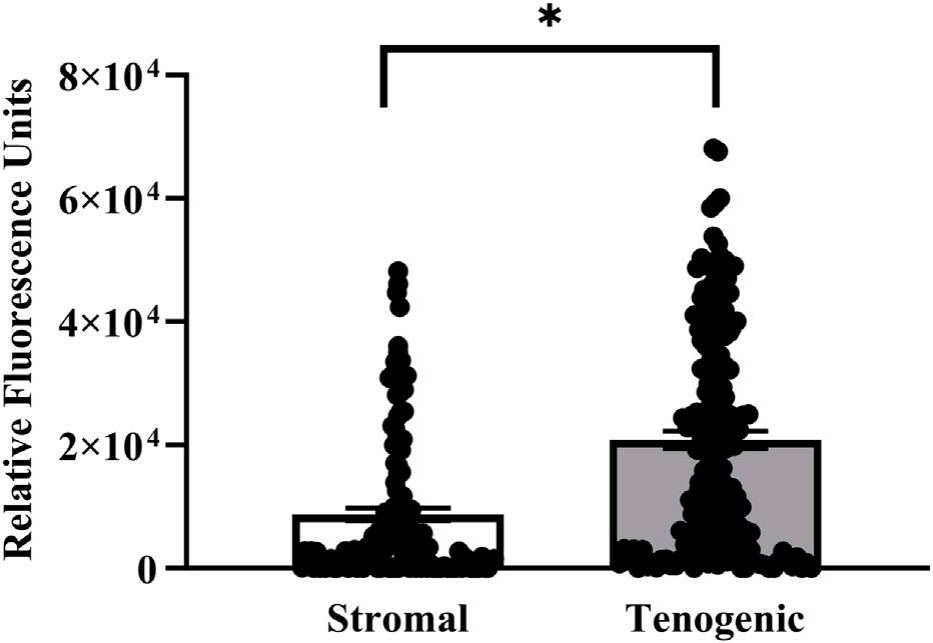
Relative viable cell number in COLI-ASC constructs. Fluorescent intensity of resorufin (mean ± SEM) in constructs cultured in stromal or tenogenic medium with all time points combined. A difference between culture medium columns is indicated by an asterisk (*p* < 0.05).

Viable cells were apparent in both stromal and tenogenic medium up to 21 days of culture, and they were present throughout the width and length of the constructs at all time points (Figure 5). They were spherical in stromal medium for all culture periods and in tenogenic medium on day 7. Many viable cells had a spindle-shape and parallel alignment in tenogenic medium after 14 and 21 days of culture. Viable cells were frequently in clusters in tenogenic medium while individual cells were prevalent in stromal medium. Differences in the relative fluorescence units among time points for each culture medium were not significant, so time points were combined. The relative fluorescence units of constructs cultured in tenogenic was significantly higher than in stromal medium with all time points combined (*p* < 0.0001, Figure 6).

### Gene expression

**FIGURE 7:**
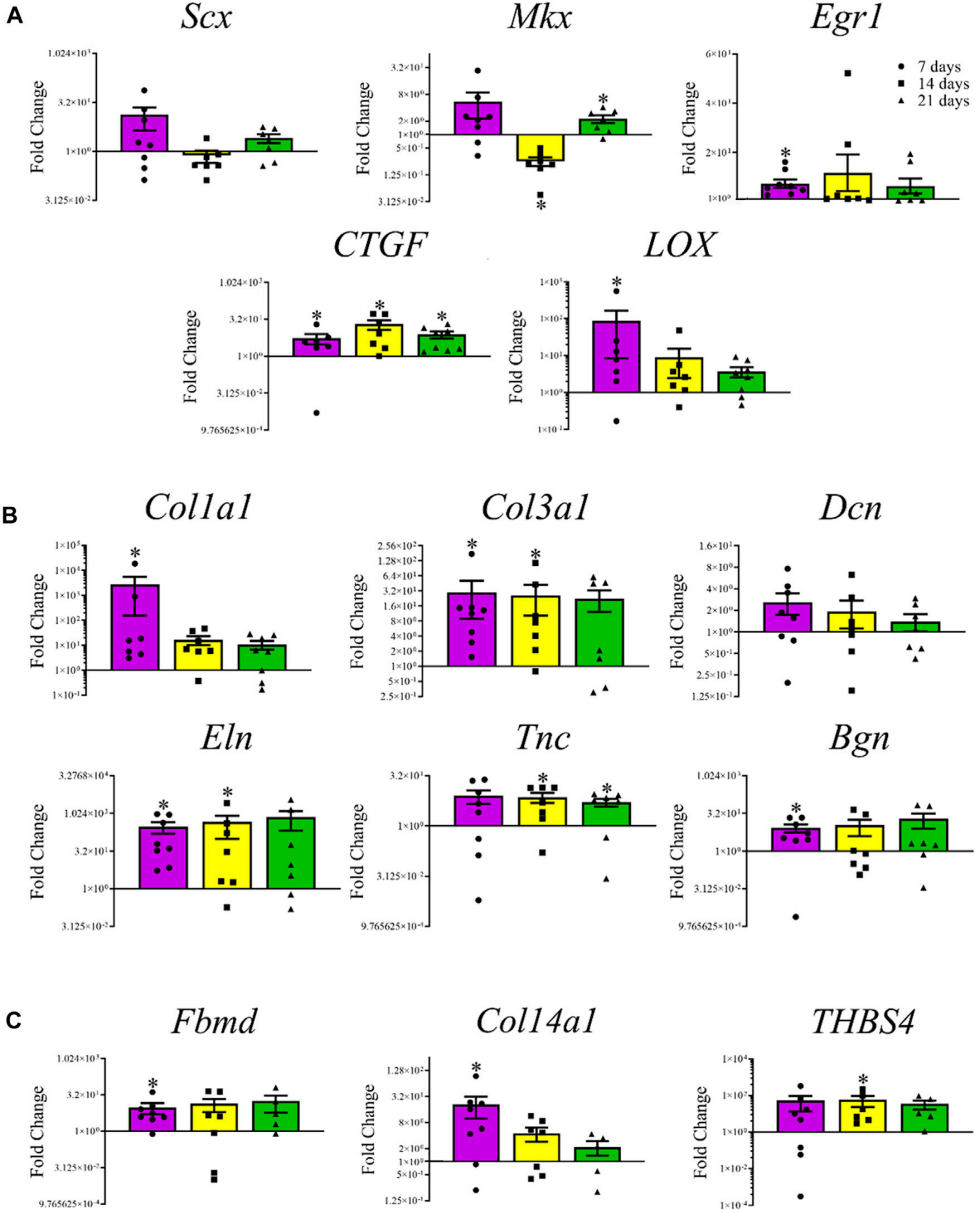
Tendon-related gene expression of construct cells. Fold change (mean ± SEM) in mRNA levels of tenogenic transcription factors *Scx*, *Mkx*, *Egr1*, *CTGF*, and *LOX* **(A)**, tenogenic ECM genes *Col1a1*, *Col3a1*, *Dcn*, *Eln*, *Tnc*, and *Bgn* **(B)**, and mature tendon markers, *Fbmd*, *Col14a1*, and *THBS4* **(C)** by cells in COLI-ASC constructs cultured in tenogenic medium relative to those cultured in stromal medium for 7 (purple), 14 (yellow), or 21 (green) days. Asterisks indicate a difference from 1-fold within each culture period.

Among the transcription factors, *Mkx* levels were lower (0.75 ± 0.05-fold, *p* < 0.0001) after 14 days and higher (2.28 ± 0.46-fold, *p* = 0.03) after 21 days, respectively, in constructs cultured in tenogenic medium (Figure 7A). The *Egr1* (7.31 ± 1.71-fold, *p* = 0.008) and *LOX* (86.96 ± 78.50 -fold, 0.03) were higher after 7, and *CTGF* after 7 (5.48 ± 2.47-fold, *p* = 0.03), 14 (20.4 ± 8.76-fold, *p* = 0.02), and 21 (7.69 ± 2.50-fold, *p* = 0.008) days of culture. Among tenogenic genes, mRNA levels of *Col1a1* (2.78 × 10^3^ ± 2.63 × 10^3^-fold, *p* = 0.02), *Col3a1* (2.98 × 10^1^ ± 2.08 × 10^1^-fold, *p* = 0.008), *Eln* (3.03 × 10^2^ ± 1.44 × 10^2^-fold, *p* = 0.008), and *Bgn* (8.52 ± 3.03-fold, *p* = 0.02) were higher after 7 days of culture; *Col3a1* (2.62 × 10^1^ ± 1.59 × 10^1^-fold, *p* = 0.03), *Eln* (4.63 × 10^2^ ± 3.65 × 10^2^-fold, 0.05), and *Tnc* (7.29 ± 2.40-fold, 0.03) were higher after 14 days of culture; and *Tnc* mRNA levels were higher after 21 (5.12 ± 1.35-fold, *p* = 0.03) days of culture (Figure 7B). The mRNA levels of mature tendon markers, *Fbmd* (9.75 ± 4.68-fold, *p* = 0.02) and *Col14a1* (2.10 × 10^1^ ± 1.1 × 10^1^-fold, *p* = 0.04) were higher after 7 days, and *THBS4* (5.85 × 10^1^ ± 3.55 × 10^1^-fold, *p* = 0.03) after 14 days of culture (Figure 7C).

### Histological analysis

**FIGURE 8:**
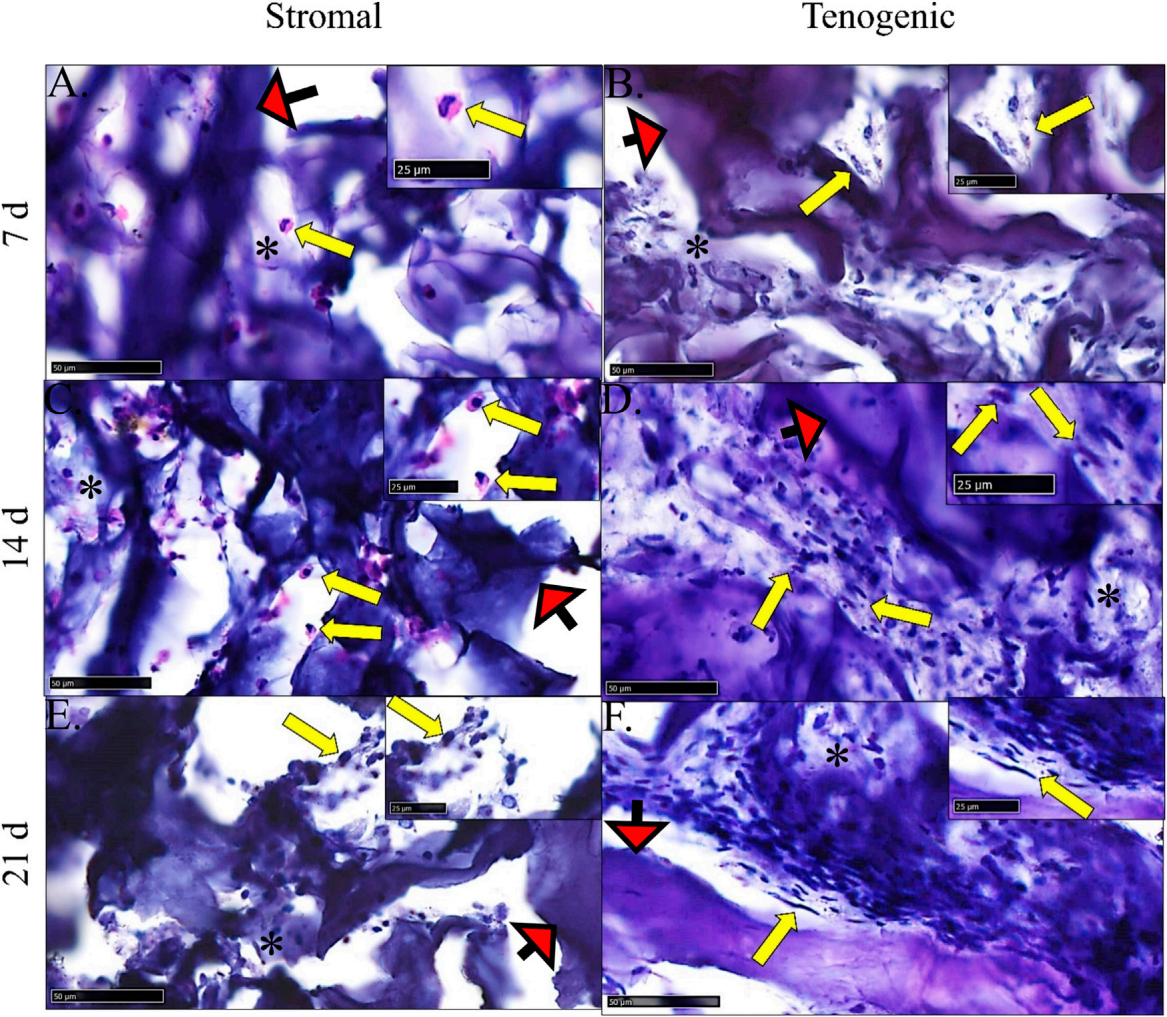
Construct histology. Photomicrographs of COLI-ASC constructs cultured in stromal **(A,C,E)** or tenogenic **(B,D,F)** medium for 7 **(A,B)**, 14 **(C,D)**, or 21 **(E,F)** days. Cells (yellow arrows) within variable amounts of ECM (*) as well as template material (red arrows) were apparent. The region surrounding each arrow is enlarged in the inset at the right upper corner. Stain: H&E; Scale bars = 50 μm **(A–C,E)**, 100 μm **(D,F)**.

There was no evidence of cell necrosis or apoptosis in constructs cultured in either medium at any time point based on intact cell and nuclear membranes and little to no cytosolic vacuolation (Figure 8). Rare, individual spheroid cells containing large nuclei, attached to template COLI fibers, and with little to no surrounding ECM were evident in constructs cultured in stromal medium for 7 and 14 days. After 21 days of culture in stromal medium, individual, or clustered, spindle- or rhomboid-shaped cells with small, dense nuclei and scant ECM were evident.

Within constructs cultured in tenogenic medium, there were clusters of spindle-shaped cells with large, round to oblong nuclei that were adhered to template COLI fibers and surrounded by ECM after 7 days of culture. After 14 days of culture, cell density and ECM were higher, most cells had a spindle-shaped morphology with dense, elongated nuclei, and cells were positioned in a more parallel arrangement compared to day 7. By day 21 of culture, closely packed, elongated cells with a dense, rod-like nucleus and surrounded by well-organized ECM were aligned in parallel with each other and template COLI fibers.

### Immunohistochemical fibromodulin staining

**FIGURE 9:**
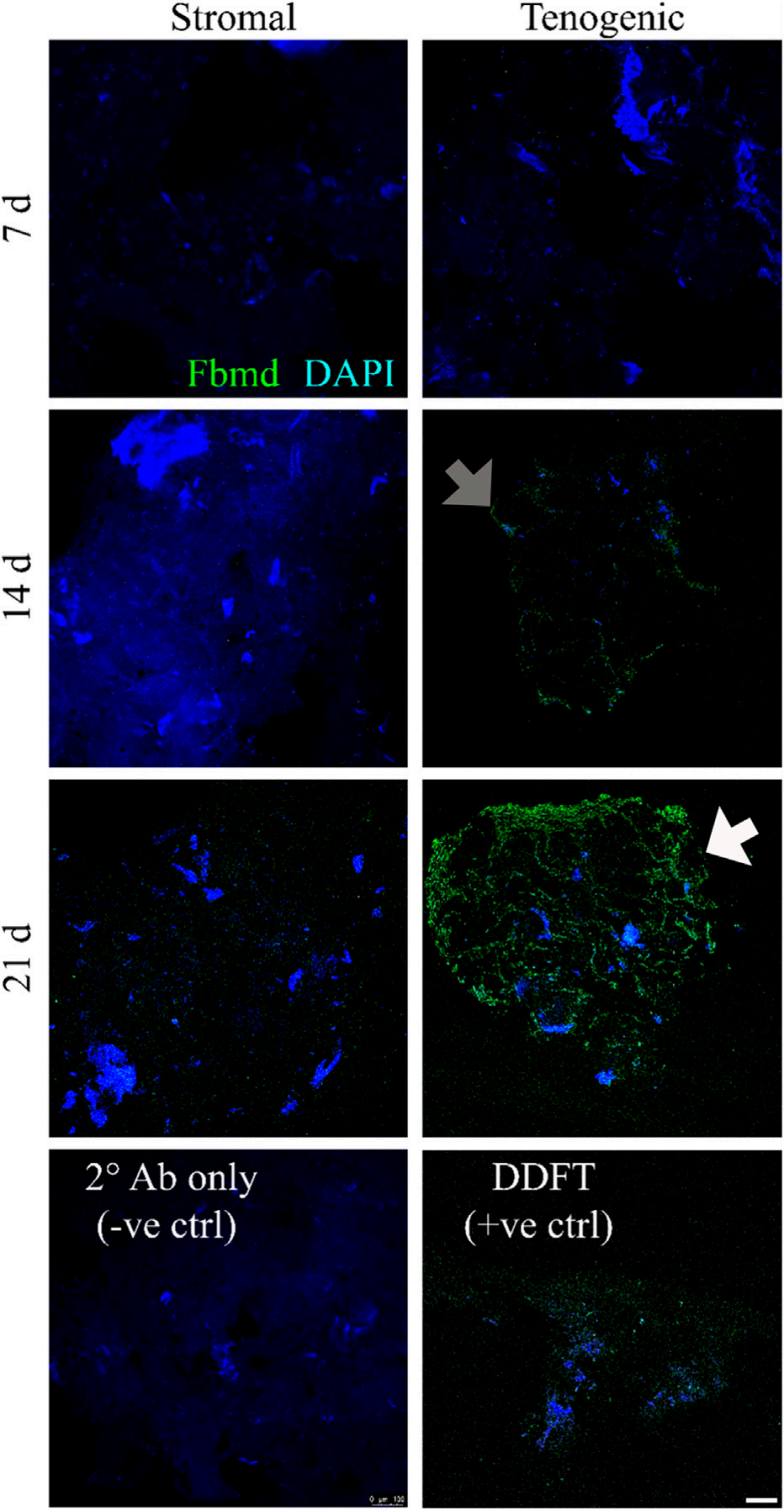
Immunohistochemical fibromodulin staining. Fibromodulin (Fbmd, green) localization in COLI-ASC constructs cultured in stromal (left) or tenogenic (right) medium for 7, 14, or 21 days, and in a section of deep digital flexor tendon (DDFT). A construct section stained with only secondary antibody (2° Ab only) is also shown. Scant, non-specific staining (gray arrow) was apparent after 14 days in constructs cultured in tenogenic medium, and, after 21 days of culture in tenogenic medium, rings of fibromodulin surrounded well-circumscribed regions within constructs (white arrow). Cell clusters (blue) were apparent in all constructs. DAPI counterstain; Scale bar = 100 μm.

There was no fibromodulin staining in constructs cultured in stromal medium for 7 or 14 days, and only scant, non-specific fibromodulin staining was present after 21 days (Figure 9). In constructs cultured in tenogenic medium, no staining was evident after 7 days, scant, non-specific staining was apparent after 14 days, and, after 21 days of culture, well circumscribed rings of fibromodulin deposition were evident within constructs. Cells within constructs were identified by nuclear staining.

### Scanning electron microscopy (SEM)

**FIGURE 10:**
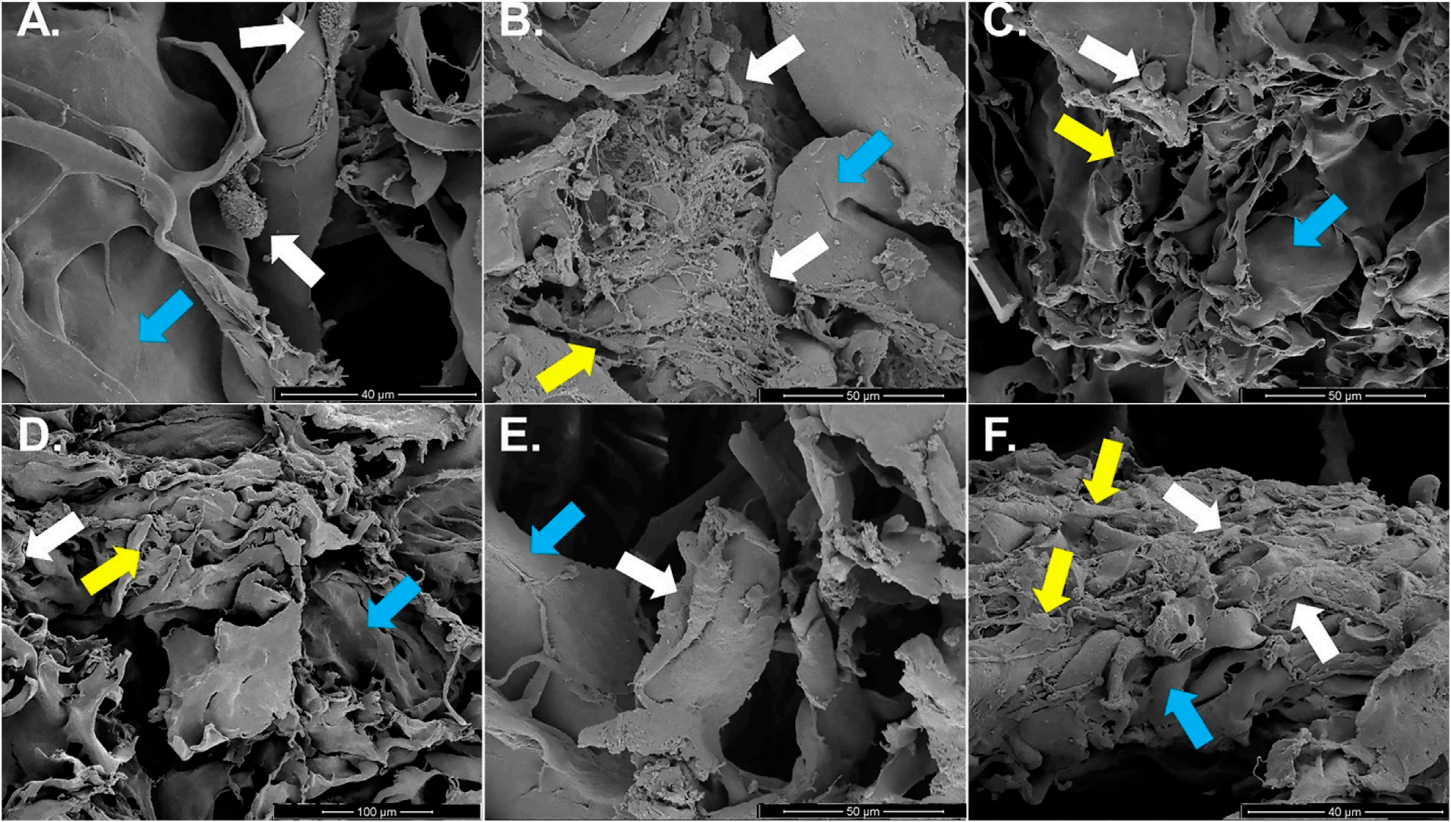
Construct surface ultrastructure. Scanning electron photomicrographs of COL1-ASC constructs cultured in stromal **(A–C)** or tenogenic **(D–F)** medium for 7 **(A,D)**, 14 **(B,E)**, or 21 **(C,F)** days with cells (white arrows), on template collagen fibers (blue arrows) and variable amounts of ECM (yellow arrows). Scale bars = 40 μm **(A,F)**, 50 μm **(B,C,E)**, 100 μm **(D)**.

Individual or clusters of round cells loosely adhered to template fibers with minimal, poorly organized ECM were present in constructs cultured in stromal medium up to 21 days (Figures 10A–C). The amount of ECM was higher after 14 and 21 versus 7 days of culture. Constructs cultured in tenogenic medium appeared to have small, round cells attached in clusters to template and minimal ECM after 7 days of culture. In contrast, elongated cells that were tightly adhered to collagen template within some ECM were apparent after 14 days, and after 21 days, numerous, rhomboid cells, covered by ECM were tightly adhered to template fibers (Figures 10D–F).

### Transmission electron microscopy (TEM)

**FIGURE 11:**
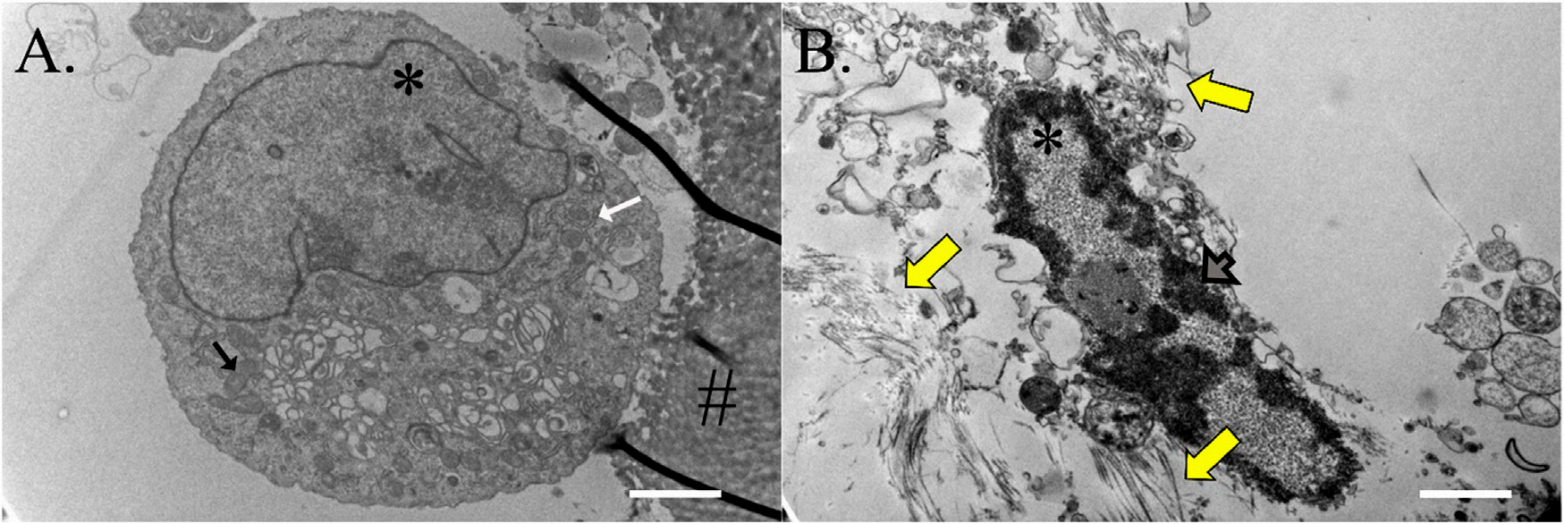
Construct cell ultrastructure. Electron microphotographs of cells from ASC-COLI constructs cultured in stromal **(A)** or tenogenic **(B)** medium for 21 days with fibrils in the ECM surrounding the latter (yellow arrows). Cells within constructs cultured in stromal medium had abundant rough endoplasmic reticulum (white arrow) and mitochondria (black arrow), and heterochromatin was apparent in the nuclei of construct cells cultured in tenogenic medium (gray arrow). Nuclei are indicated with an asterisk (*) and template material with a hash (#). Scale bars = 2 μm **(A)**, 1 μm **(B)**.

Cells on ASC-COLI constructs cultured in stromal medium for 21 days were round, had a high nucleus to cytosol ratio, and were rich in mitochondria and rough endoplasmic reticulum (Figure 11A). Some were attached to template COLI fibers. Cells on constructs cultured in tenogenic medium were oval and had minimal cytoplasm surrounding a rod-shaped nucleus with abundant heterochromatin (Figure 11B). Fibrils were present in the surrounding ECM.

## Discussion

The results of this study confirm that culture in custom bioreactors with combined perfusion and centrifugal tenogenic medium circulation supports differentiation of equine adult ASCs into tendon progenitor-like cells capable of neotissue formation. Viable cells were present throughout constructs cultured in stromal and tenogenic medium for up to 21 days of culture. However, viable cell numbers, gene expression, fibromodulin localization, micro- and ultrastructure, were distinct between culture media. The hypothesis that ASCs assume a tendon progenitor cell-like morphology, express tendon-related genes, and produce more organized extracellular matrix in tenogenic versus stromal medium with perfusion and centrifugal fluid motion was accepted. *De novo* generation of viable tendon neotissue using the methods described has the potential to contribute to novel tendon therapies for multiple species.

Collagen-based templates to support MSC differentiation come in many forms. Hydrogels, decellularized tendon tissue, and various sponge configurations are among the most common (Noth et al., 2005; Theiss et al., 2015; Clements et al., 2016; Youngstrom et al., 2016). In general, cell distribution in COLI sponge is more challenging than in the stable architecture of decelluarized tissue or malleable, semi-solid hydrogels with low fiber density and stiffness. When COLI sponge is hydrated, the fibers adhere to each other, which reduces porosity and can interfere with cell migration and gas and nutrient exchange. Use of culture mechanisms with dynamic fluid flow are designed to amelioriate these limitations. Shear stresses and better gas and nutrient delivery from centrifugal fluid motion generated with a stir bar (50 rpm) reportedly improves MSC proliferation on collagen sponges reinforced with polyethylene terephthalate compared to no fluid flow (Takamoto et al., 2012). The benefits of dynamic perfusion are similar to those of centrifugal forces, but the shear forces are typically normal, or aligned with the long axis of the construct, versus the tangential shear forces of centrifugal fluid flow (Xie et al., 2013; Duan et al., 2017; Taguchi et al., 2021). Centrifugal and perfusion fluid flow were uniquely combined in the custom bioreactors within this study. Perfusion through the medium reservoir ensured adequate medium gas exchange during long term culture, and the normal and tangential shear forces provided unique mechanical stimulation. It is possible that the combined forces improved fluid motion within the construct, though this was not measured directly. Damage to the constructs cultured in stromal medium in this study was likely a result of shear forces that were highest near the stir bar. The effect was not apparent in constructs cultured in tenogenic medium. This could have been from better tissue stability from abundant, organized ECM. The magnitude of dynamic perfusion and centrifugal fluid motion will need to be customized for bioreactor and template configuration and size.

Tenogenic differentation is a result of cross talk between chemical and mechanical signals, and neotissue is defined as a combination of cells and the proteinaceous extracellular they produce (Mace et al., 2016; Mehrian et al., 2018). Construct strain and shear fluid forces combined with tenogenic medium were designed to drive ASC differentiation and generation of tendon neotissue in this study. Current knowledge supports that construct strain is imperative to effective tenogenic differentiation of progenitor cells, and variations in static or dynamic strain affect cell proliferation and the rate and efficiency of tendon neotissue generation (Mace et al., 2016; Engebretson et al., 2017; Mehrian et al., 2018; Zhang et al., 2018). During culture, cells align with the direction of construct strain (Mozdzen et al., 2016). The maintenance of a construct strain of 10% in this study likely promoted cell distribution and alignment along tensioned COLI fibers, and may have supported cell proliferation (Kuo and Tuan, 2008; Mozdzen et al., 2016; Engebretson et al., 2017). However, the culture conditions alone were not sufficient to drive cell differentiation and ECM deposition without medium originally designed for equine tenocyte culture (Theiss et al., 2015). Combined with the other medium components, TGF-β is a vital growth factor for differentiation of progenitor cells into tenocytes. Differentiation is regulated by TGF-β/Smad2/3 signal transduction, and TGF-β also promotes collagen production by the cells (Y. Li et al., 2022). The authors, acknowledge, however, the presence of growth factors that were not quantified in the FBS that was used in both culture media. Further refinement of strain and culture conditions to produce tendon neotissue on equine ASC-COLI constructs could improve efficiency to scale up production (Engebretson et al., 2017; Grier et al., 2017; Mozdzen et al., 2016; Taguchi et al., 2021; Youngstrom et al., 2016).

Within the acknowledged limits of the RT-PCR methods used in this study, the change in gene expression profile of cells within constructs cultured in tenogenic medium over the 21 day culture period supports early cell differentiation to a tenogenic lineage. Early, high expression of *Scx, Mkx, Egr1*, *CTFG,* and *LOX,* transcription factors essential for initiation of progenitor cell tenogenic differentiation, suggests that the differentiation process began early in the culture period at the genetic level (Derby et al., 2012; Sakabe et al., 2018; Korcari et al., 2022). Lower expression of Mkx after 14 days of culture could have been due to a smaller population of undifferentiated cells compared to after 7 days, while, due to cell proliferation, there was a larger population of undifferentiated cells after 21 versus 14 days. Since *CTFG* is a constitutively expressed tenogenic transcription factor that inhibits maturation of tenogenic progenitors, highest expression after 14 days followed by 21 days of culture indicates cell immaturity and suggests proliferation capabilities (Chen et al., 2008; Li et al., 2019; Rui et al., 2019). Given that LOX enzymes play a role in collagen crosslinking that occurs during embryonic tendon development, decreases in LOX mRNA levels with culture time suggest some progression in ECM maturity (Marturano et al., 2014; de Aro et al., 2018; Pan et al., 2018; Ellingson et al., 2022).

The ECM gene mRNA levels in constructs cultured in tenogenic medium support deposition and subsequent organization of fibrous ECM observed with light and electron microscopy. Higher levels of *Col1a1* RNA initially may be related to the differentiation process, after which stable levels of *Col1a1* and *Col3a1* at a ratio characteristic of healthy versus abnormal tissue deposition were maintained (de Girolamo et al., 2015; Stanco et al., 2019). Tropoelastin is important for adult tendon function, though less is known about its role in tendon development (Brown et al., 2015; Halper, 2021). Tenacins contribute to coordinated generation and repair of musculotendinous tissue, and decorin and biglycan are small leucine-rich proteoglycans that are critical to collagen fibrillogenesis for tendon development (Taylor et al., 2009; Dyment et al., 2013; Leiphart et al., 2020; Halper, 2021).

Genes associated with mature tendon tissue evaluated in this study, *Fbmd, Col14a,* and *THBS4*, are also associated with embryonic tendon formation. Specifically, *Fbmd* is upregulated in tendon tissue during embryogenesis and during the neonatal period, *Col14a1* gene and protein levels are elevated in neonatal tissue, and *THBS4* is highly expressed in ovine calcaneal tendon during early gestation and gradually decreases with age to low levels in adults (Ansorge et al., 2009; Dyment et al., 2013; Liu et al., 2012; Russo et al., 2015). Gene expression results are relative and limited to the comparisons between culture conditions in this study. Taken together, the transcription factor, extra-cellular matrix, and mature tendon gene expression profiles suggest tenogenic differentiation of ASCs on COLI constructs cultured in tenogenic medium relative to those cultured in stromal medium.

Changes in cell and ECM morphology paralleled gene expression and were consistent with tenogenic differentiation (Chen et al., 2014; Schulze-Tanzil et al., 2004). A different appearance, greater adhesion between construct layers, and firmer texture of constructs cultured in tenogenic medium are consistent with tenogenic differentation and aligned with the more highly organized, fibrillar ECM visible at the micro- and ultra-structural levels. The cell morphology and rudimentary ECM evident in constructs cultured in stromal medium could represent some level of differentiation since COLI-based matrix, with and without strain, is reported to induce progenitor cell differentiation (Cardwell et al., 2014; Grier et al., 2017; Grier et al., 2019; Kim et al., 2015). Transmission electrophotomicrographs highlighted distinct cell maturities. Specifically, those in tenogenic medium had the appearance of differentiated cells surrounded by collagen-like fibrils, while those in stromal medium had the appearance of immature progenitor cells (Schneider et al., 2011; Ugarte et al., 2015). Immunolocalization of fibromodulin, a bioactive factor of native tendon ECM suggested to be a differentiation marker of tendon, additionally supports the presence of tendon progenitor cells in the constructs cultured in tenogenic medium (Miyabara et al., 2014; Ning et al., 2021). The protein is a vital component of the tendon progenitor cell ECM niche that controls tendon progenitor cell self-renewal and differentiation (Bi et al., 2007). It is also thought to regulate collagen fibril size by controlling premature cross-linking via LOX modulation (Taye et al., 2020). The pattern of fibromodulin labeling in the constructs cultured in tenogenic medium resembled that of mature equine where the strongest staining is in the intrafasicular matrix surrounding bundles of collagen called fascicles (Thorpe et al., 2016; Taye et al., 2020). Lower, poorly organized labeling in constructs cultured in stromal medium highlights some low level of cell differentiation as mentioned above. The gross appearance, micro- and ultrastructure, and immunohistochemical labeling of constructs cultured in tenogenic medium are consistent with early tendon neotissue.

The authors acknowledge limitations to this *in vitro* study. Constructs were surrounded by suture in a pattern that allowed strain to be applied to them within the bioreactor chamber as previously reported. The suture may have impacted shear forces on the construct surface and cell migration from compression of construct material directly beneath suture strands. A mechanism to apply strain without focal compression could potentially improve cell movement and consistency in shear forces. Similarly, centrifugal forces were not identical along the length of the construct due to the position of the stir bar at the lowest end of the bioreactor. As previously mentioned, higher forces at the lower end of the construct may have damaged constructs cultured in stromal medium. The method for RNA isolation from the constructs included collagenase digestion like descriptions of RNA isolation from fibrous tissues (Burja et al., 2022; Grinstein et al., 2018; Takacs et al., 2023). The potential that RNA quantity was compromised cannot be entirely ruled out, however. Comparisons in this investigation are limited to the specific primary cell isolates and culture conditions described here.

The unique combination of static strain and centrifugal and perfusion fluid flow of tenogenic medium supported *de novo* tendon neotissue from adult equine ASCs. *De novo* equine tendon neotissue tissue production could be a useful resource for investigations surrounding tendon formation and pathology. This is especially important to reduce animal use for screening therapeutic compounds and technology. Injectable, collagen- and lyophilized tendon-based hydrogels with and without progenitor cells have been explored for treatment of tendon pathology (Crowe et al., 2016; Liu et al., 2020). Ultrasound is the established imaging modality to identify and monitor healing of equine tendon lesions, and injection of cell- and cell product-based therapies into equine tendon lesions with ultrasound guidance is a contemporary standard of care (Dyson, 2004; Geburek et al., 2017; Giunta et al., 2019; Romero et al., 2017; Witte et al., 2016). Tendon neotissue stiffness is compatible with injection, so it is feasible that it could be administered with ultrasound guidance following appropriate safety and efficacy testing. Taken together, the unique culture system and methods of this investigation have the potential expand the current repertoire of adult multipotent stromal cell-based products to the neotissue level.

## Data Availability

The original contributions presented in the study are included in the article/supplementary material, further inquiries can be directed to the corresponding author
